# Synthesis, molecular simulation studies, in vitro biological assessment of 2-substituted benzoxazole derivatives as promising antimicrobial agents

**DOI:** 10.55730/1300-0527.3535

**Published:** 2022-12-29

**Authors:** Gajanan S. SHANBHAG, Amit BHARGAVA, Giridhar PAL SINGH, Shrinivas D. JOSHI, Narendra SINGH CHUNDAWAT

**Affiliations:** 1 Department of Chemistry, Bhupal Nobles’ University, Udaipur, Rajasthan, India; 2 Department of Pharmacy, Bhupal Nobles’ University, Udaipur, Rajasthan, India; 3 Department of Pharmaceutical Chemistry, S. E. T’s College of Pharmacy, Sangolli Rayanna Nagar, Dharwad, Karnataka, India

**Keywords:** Benzoxazole, antimicrobial activity, molecular docking, DNA gyrase, greener synthesis

## Abstract

The 2-substituted benzoxazole derivatives are known to exhibit a wide spectrum of biological potential. Two series of novel benzoxazole derivatives containing 2-phenyl and 2-N-phenyl groups were synthesized, by following the green chemistry approach. All the newly synthesized derivatives were screened against gram-positive bacteria (*Streptococcus pyogenes*, *Staphylococcus aureus*), gram-negative bacteria (*Pseudomonas aeruginosa*, *Escherichia coli)* and the fungus (*Aspergillus clavatus and Candida albicans)*. Most of these compounds have demonstrated potent antibacterial activities, especially against *E. coli* at 25 μg/mL, along with moderate antifungal activity. Among these, two compounds, 21 and 18, showed interesting antibacterial profile. Molecular docking studies suggested that the antibacterial activity can be linked to the inhibition of *DNA gyrase*. Overall, the study proposes that these biologically potent compounds can be considered for developing the next generation antimicrobial agents.

## 1. Introduction

The emergence and spreading of drug-resistant pathogens with newer resistance mechanisms, are leading to antimicrobial resistance, which threatens existing drugs’ ability to treat common infections. Invention of the new chemical entities to deal with resistant bacteria and fungi has become one of the major challenges in the research areas of antibacterial and antifungal discovery. This is prompting academics to come up with novel and potent antibacterial as well as antifungal therapeutic agents [1].

The active heterocyclic compounds are one of the main topics of interest for medicinal chemists as it is proven to display various pharmacological activities. Heterocyclic compounds containing nitrogen, oxygen, and sulphur belonging to five or six membered heterocycles have gained enormous significance, due to their interesting and diverse clinical applications in the field of medicinal chemistry [2]. Oxazoles are the five membered heterocycles containing nitrogen and oxygen with wide range of pharmacological properties. Benzoxazole belongs to the class of heterocycles, containing a bicyclic structure in which the oxazole moiety is fused with the aryl ring. Benzoxazole and their derivatives are important scaffolds often found in the various drugs and pharmaceutically relevant compounds [3]. They exhibit wider range of biological activities like antifungal [4], antituberculosis [5], anticancer [6], antiinflammatory-analgesic [7], antitumor [8], and antibacterial [9]. Increasing applications of benzoxazole derivatives have intrigued many researchers to come up with several benzoxazole derivatives and their evaluation as pharmaceutical agents [10].

Among the benzoxazole derivatives, the 2-substituted benzoxazole has always attracted many researchers owing to its diverse applications in medicinal chemistry [11]. These 2-substituted benzoxazole containing scaffolds demonstrated various pharmacological activities such as antiviral [12], antimicrobial [13], and anticancer [14], properties. Recently, we have demonstrated the antimycobacterial potential of 2-substituted benzoxazole sulphonamides [15].

Knowledge on the mechanism of action and target identification in early stage of the drug discovery process provides distinctive benefits. Identifying a drug’s mode of action (MOA) could increase the probability of success of clinical trials [16]. Molecular docking technique plays a key role in identifying a drug’s mode of action. In the molecular docking studies, small molecules are docked into the receptor targets in order to predict bioactive confirmation of the ligand and its orientation inside a target binding site [17].

**Figure 1:**
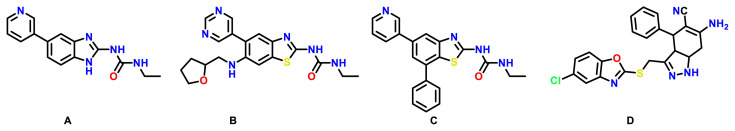
Known inhibitors of DNA gyrase containing benzoxazole, benzimidazole, and benzothiazole moiety.

Over the years, scaffold hopping has been a common medicinal chemistry approach in finding effective inhibitors including DNA gyrase. Using this approach, bicyclic ring structures like benzimidazole, benzothiazole [18, 19, 20], and benzoxazole [21] have been tested as inhibitors of DNA gyrase (Figure 1). Particularly Oksuzoglu et al*.* synthesized antibacterial 2-benzyl substituted benzoxazole derivatives. Antibacterial potentials of these compounds were linked to DNA topoisomerase II inhibitors with significant IC_50_ values [22].

DNA gyrase is an essential enzyme and a part of topoisomerase which is responsible for catalysing the changes to DNA topology. The existence of DNA gyrase in all bacteria and absence of the same in higher eukaryotes makes it an attractive drug target for antibacterial studies [23].

**Figure 2:**
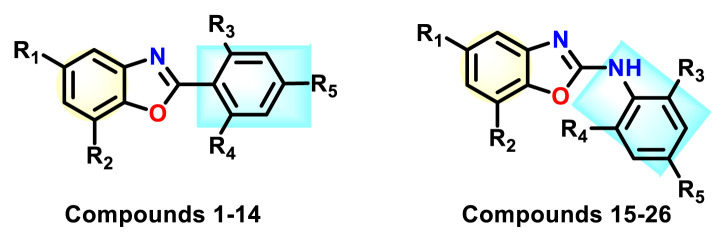
Compound library of 2-phenyl and N-phenyl benzoxazole-based scaffolds.

Having the initial understanding on benzoxazole derivatives and utilising the interplay of conventional techniques of structure-based design and docking, in the present work, we have synthesized a small library of 2-phenyl-1,3-benzoxazoles (compounds 1–14) and N-phenyl-1,3-benzoxazol-2-amine (compounds 15–26) (Figure 2.) These compounds were evaluated for their antimicrobial properties. Further based on the biological outcome, structure activity relationship (SAR) was established. Molecular modelling and docking simulation studies were executed to get the insights on potent antimicrobial action of the synthesized derivatives, in the binding sites of DNA gyrase of *E. coli*.

Targeted compounds were synthesized using an environmentally benign process. 2-phenyl substituted benzoxazole (compounds 1–14) were synthesized using green catalyst “fly ash” and N-phenyl-1,3-benzoxazol-2-amine derivatives (compounds 15–26) were synthesized utilising less expensive, I_2_-mediated oxidative cyclodesulfurization.

## 2. Materials and methods

### 2.1. General information

All reagents and solvents were purchased from commercial sources and used without further purification. Analytical grade solvents were utilised in this work and used as received. All the reactions were carried out using magnetic stirring, using appropriate conditions, and using oven-dried glassware. Reactions were monitored by thin layer chromatography (TLC) using TLC silica gel plates and visualizing agent as UV light. Reaction products have been purified either by recrystallization in analytical grade ethanol or by column chromatography using Merck silica gel (230–400 flash). Bruker supercon magnet avance DRX-300 spectrometer was utilised for NMR spectra recording. 300 MHz spectrometer was used for ^1^H NMR and 100 MHz for ^13^C NMR recording in deuterated solvents with TMS as an internal reference standard. Chemical shifts *δ* have been given in ppm, and coupling constant *J* in Hz. Recorded multiplicities are shown as follows: singlet (s), doublet (d), triplet (t), broad singlet (br s), and multiplet (m). Mass spectra were taken in either ESI positive or negative ion mode. Characterization of all compounds was done by TLC, ^1^H NMR and ^13^C NMR, IR and MS.

### 2.2. General experimental method for the synthesis of substituted 2-aryl benzoxazoles (compounds 1–14)

Step 1: Preparation of preheated fly ash (PHFA)

The fly-ash obtained from commercial source was heated in hot air oven up to a temperature of 110 °C for 2 h. Heating process helps to avoid colloidal formation during the reaction due to demoisturization. Thus, obtained activated form of fly ash was directly used for the next step.

Step 2: Common procedure for synthesis of compounds 1–14

Appropriate mixture of an aldehyde (1.1 mmol) (1), *o*-aminophenol (2) (1.0 mmol) and preheated fly-ash (0.5g) were taken in 50 mL of RB flask under nitrogen. The mixture was stirred at 111 °C in the presence of 20 mL of toluene. After the reaction completion by TLC (starting material disappearance), the reaction mass was cooled to room temperature. Reaction mixture was filtered using hyflow bed and washed with ethyl acetate to remove the catalyst. Combined organic layer was evaporated under reduced pressure. The crude material was purified using either column chromatography ethyl acetate: hexane (1:9) or recrystallization with ethanol to isolate the pure product (compounds 1–14) in 70%–85% yields.

### 2.3. Common experimental procedure for the synthesis of substituted N-phenyl-1,3-benzoxazol-2-amine derivatives (compounds 15–26)

To a prestirred solution of aminophenol (4, 1 mmol) in THF (5 mL), corresponding isothiocyanate (5, 1.1 mmol) was added slowly at the room temperature. After the absence of the aminophenol (as indicated by TLC), iodine (0.6 mmol) and K_2_CO_3_ (1.1 mmol) were added in sequence. The reaction mixture was stirred at ambient temperature, until TLC indicates the disappearance of the in situ generated intermediate D (Scheme 2). After the completion of the reaction, it was quenched with aqueous 5% Na_2_S_2_O_3_ solution (5 mL), and diluted with brine (10 mL) solution. Reaction mass was then extracted with ethyl acetate (20 mL* 2). The pooled organic layers were dried over anhydrous sodium sulphate, concentrated under vacuum which was further purified either by recrystallization or by silica gel column chromatography using a mixture of ethyl acetate/hexane (2:8) as the eluent to afford the product (compounds 15–26) ranging from 65% to 89% yields.

Compounds characterization data can be found in supporting information.

### 2.4. Antimicrobial studies

Whole-cell growth inhibition assay was used for antimicrobial screening. All the microbial strains were obtained from the microbial type culture collection and gene bank (MTCC), Chandigarh, India. All the synthesized benzoxazole derivatives were screened to check their in vitro antibacterial potential. Based on their clinical and pharmacological importance of the strains, compounds were screened against gram-positive (*Streptococcus Pyogenes* and *Staphylococcus aureus*), gram-negative (*Pseudomonas aeruginosa* and *Escherichia Coli*) and two fungal strains (*Aspergillus clavatus* and *Candida albicans*) by means of agar diffusion method [24].

#### 2.4.1. Antibacterial activity

Using spread plate technique, all the bacterial strains were cultured on YEPD or nutrient agar at 37 °C. Bacterial strains were maintained on nutrient agar slants at 4 °C. Different concentrations of the test compounds (5, 25, and 50 μg/mL) along with standard drug cefixime (positive control) were prepared to execute antimicrobial sensitivity. Compound susceptibility was measured for different strains of bacteria on the basis of zone of inhibition diameter in comparison with the standard drug [25]. Initial results indicated that 5 μg/mL produced less desired inhibitory effect and the dose of 50 ug/mL showed more effective antimicrobial action of substance than the desired. Hence, 25 μg/mL dose was used further for the present research work. The dilutions (25 μg/mL) of benzoxazole derivatives and positive control drug were prepared in DMSO and double-distilled water using tubes of nutrient agar. Mueller-Hinton sterile (MHA) agar plates were seeded with different bacterial strains (108 cfu) and incubated at 37 °C for 18 to 24 h and the zones of growth inhibition around the disks were calculated.

#### 2.4.2. Antifungal activity

Screening of antifungal compounds was carried out against *A. clavatus* and *C. albicans* using agar disk diffusion method. Stock of fungal strain cultures were allowed to incubate for 24 h at 37 °C and then at refrigeration storage of 4 °C. The yeasts were grown in sabouraud dextrose agar (SDA) and the molds were grown on PDA media at 28 °C. Zone of inhibition was measured in the similar way as in the case of antibacterial activity after 48 to 96 h using griseofulvin (standard drug) (25 μg/mL) as a positive control.

In both the cases, the microorganism species sensitivities to the test and control sets were determined by assessing the sizes of inhibitory zones (the diameter of disk) around the disks on the agar surface, and values of <8 mm were considered not active.

#### 2.4.3. Minimum inhibitory concentration (MIC) [26]

The benzoxazole derivatives which demonstrated the antimicrobial (bacterial and fungal strains) activity were later taken for minimal inhibitory concentration (MIC_50_) determination. All four bacterial strains and two fungal strains were grown in nutrient broth for 6 h. Next, 100 μL of 10^6^ cells/mL was cultured in nutrient broth tubes containing several concentrations (0.5–50 μg/mL) of selected benzoxazole derivatives. After incubation of 24 h at 37 °C, MIC value was determined for each sample, using optical density (OD) reading in the spectrophotometer (620 nm). Obtained reading was matched with control sample data of noninoculated blank nutrient broth.

### 2.5. Docking simulations

Molecular docking studies were carried out in order to understand the possible ligand-receptor intermolecular interactions. Novel benzoxazole derivatives were subjected to dock in the active site of DNA gyrase enzyme using a fully automatic docking tool, Surflex-Dock module, available on Sybyl X-2.0 version (Tripos Inc.) [27]. Binding affinities were estimated for all the compounds using the docked module. Utilising the molecular docking simulations, theoretical binding mode for all the 26 ligands at the chlorobiocin binding site was examined. To start with docking procedure, X-ray crystal structure of DNA gyrase, *E. coli* 24 kDa domain in complex with clorobiocin [28] (http://www.rcsb.org; PDB code: 1KZN; resolution 2.30A) was downloaded from the protein data bank in PDB format. Before using the protein structure for docking simulations, all water molecules were removed. In the pdb file, mislabelled atom types were adjusted; consequently, angles of proline F were anchored at 70° side chain amides and checked to maximize possible hydrogen bonding. Essential hydrogens were added and side chains were tested for close van der Waals contacts. The model was tested for conformational glitches using the ProTable module from Sybyl. Ramachandran plot of the backbone torsion angles psi and phi, the location of exposed nonpolar residues/buried polar residues and local geometry were inspected. Using Kollman united force field with dielectric constant set at 4.0 and nonbonding cut-off set at 9.0, the protein was exposed to energy minimization following gradient termination of the Powell method of 3000 iterations. Using tripos force field and Gasteiger charge with nonbonding cut-off set at 9.0 and the dielectric constant set at 4.0, energy minimization for all the synthesized derivatives and standard chlorobiocin, were carried out by Powell method for 3000 repetitions. In order to favour the binding affinity, in sybyl software using the surflex-dock programme, all the synthesized compounds were docked to DNA gyrase subunit A (PDB code: 1KZN) by incremental construction approach of building the structure in the active site. In order to have the final conclusion, various scoring functions were considered. All the docked ligands were graded based on the single consensus score (C-score).

## 3. Result and discussion

### 3.1. Chemistry

**Scheme 1:**
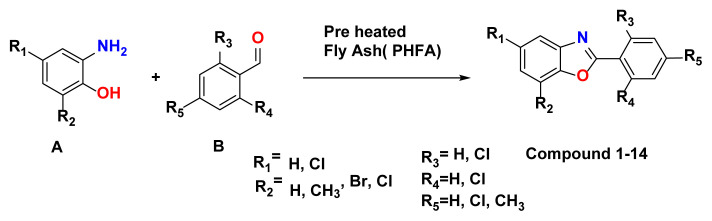
Synthesis of Aryl benzoxazoles using PHFA (preheated fly ash).

The literature reveals that there are generally two types of reactions used to construct 2-aryl benzoxazoles (compounds 1–14). One method involves transition metal catalysed intramolecular cyclisation of o-haloanilides [29], another is the condensation of 2-aminophenol with either aromatic aldehyde [30], or carboxylic acid derivatives [31] under strong oxidative conditions. These methods often suffer from many drawbacks such as the use of strongly acidic conditions, use of toxic catalysts, longer reaction time, lower product yield, use of solvent and reagents in large excess. To overcome these drawbacks, major efforts have been made for the development of environmentally benign new synthetic strategies with lesser impact on the environment. Tremendous progress has been made in synthesis of benzoxazoles by condensation method, but still there is a scope for development of environmentally benign synthetic approach by using highly efficient catalysts. Herein we report, environmentally benign synthesis of 2-phenyl substituted benzoxazoles (compounds 1–14) starting from 2-amino phenol and substituted aldehydes using green catalyst “fly ash”. (Scheme 1). In order to check the feasibility of the planned reaction methodologies, we have explored a variety of substituted aldehydes and amino phenol handles containing both electron-withdrawing and electron donating functional groups.

Scheme 1 describes the synthetic outlines for novel 2-substituted benzoxazole derivatives (compounds 1–14). Detailed synthetic procedures of 2-phenyl substituted benzoxazole derivatives (1–14) can be found in the materials and methods section.

Compounds synthesized under Scheme 1 are shown below with reaction time (h) and yield (%) data. (Table 1)

**Scheme 2:**
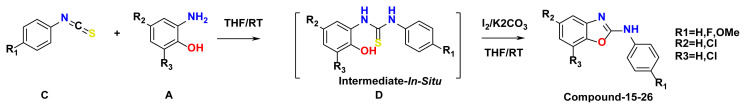
Synthetic outlines for the synthesis of substituted N-phenyl-1,3-benzoxazol-2-amine derivatives.

Synthetic procedure for N-phenyl-1,3-benzoxazol-2-amine derivatives involves in situ I_2_-mediated oxidative cyclodesulfurization (Scheme 2). In the literature, there are various approaches for the synthesis of N-phenyl-1,3-benzoxazol-2-amine derivatives (compounds 15–26). One of the common methodology to synthesise N-phenyl-1,3-benzoxazol-2-amine derivatives involve the cyclodesulfurization reaction of in situ generated monothioureas by the reaction of bis-nucleophiles such as aminophenol or O-phenylenediamine derivatives. Most common desulfurization reagents used in the past were BOP reagent [32], 1,10-(ethane-1,2-diyl) dipyridinium bistribromide (EDPBT) [33], hypervalent iodine(III) reagents [34], TsCl/NaOH [35], polymer-supported carbodiimide [36], LiOH/H_2_O_2_ [37], and HgO [38]. Most of these methodologies encounter one or other limitations such as high cost reagents or use of highly toxic reagents. Some conversions demand harsh reaction conditions or long reaction times with low chemo selectivity and yields. Herein, we describe synthesis of N-phenyl-1,3-benzoxazol-2-amine derivatives utilising less expensive and I_2_-mediated oxidative cyclodesulfurization. In order to check the feasibility of the planned reaction methodologies, we have explored a variety of substituted isothiocyanate, aminophenol handles containing both electron-withdrawing and electron-donating functional groups.

Synthetic outlines for the synthesis of substituted N-phenyl-1,3-benzoxazol-2-amine derivatives are shown in Scheme 2 (compounds 15–26). Detailed synthetic procedures of 2-N-phenyl substituted benzoxazole derivatives (15–26) are given in the materials and methods section.

Compounds synthesized under Scheme 2 are shown below with reaction time (h) and yield (%) data (Table 2).

The current methodology brings several advantages such as use of an environmentally benign process and single step synthesis with good yields (70% to 80%). All the 2-substituted benzoxazole derivatives **1–26** from both the series were found to be air and light stable.

### 3.2. Proposed mechanism

#### 3.2.1. Synthesis of 2-Aryl benzoxazoles (compounds 1–14)

**Figure 3:**
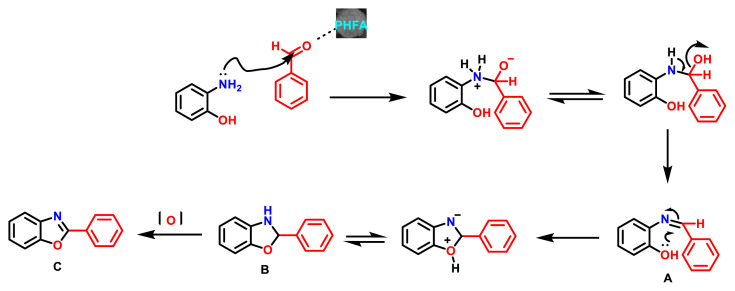
Plausible mechanism for fly ash-catalysed benzoxazole formation.

Mechanistic aspects of synthesising 2-aryl benzoxazole (C) using environmentally benign process are outlined in Figure 3. Fly ash is a by-product, resulting from the combustion of pulverized coal in the form of fine powder. It is a waste air-pollutant. Fly ash is composed of many chemical species [39]. Main contents of the fly ash are oxides of metals, namely MgO, CaO, Fe_2_O_3_, SiO_2_, Al_2_O_3_, and insoluble residues [40]. Waste fly ash can be converted into its activated form by heating in a hot air oven for 2 h at 110 °C. The chemical residues, such as oxides of mixed metals (MgO, CaO, Fe_2_O_3_, SiO_2_, and Al_2_O_3_), are present in the fly ash, which enhances the catalytic activity. The proposed general reaction mechanism is shown in Figure 3. The preheated fly ash in all probability acts as a solid catalyst which assists in the activation of aldehyde and the subsequent dehydration to form an intermediate imine (A). In the presence of O-hydroxyl group, intermediate imine (A) will undergo cyclization to produce 2-substituted-2, 3-dihydro-benzoxazole (B), which will finally get converted to the required 2-Aryl benzoxazole (C) via aerial oxidation. Literature supports similar mechanism for this type of condensation in the presence of other catalysts [41, 42, 43].

#### 3.2.2. Synthesis of N-phenyl-1,3-benzoxazol-2-amine derivatives (compounds 15–26)

**Figure 4:**
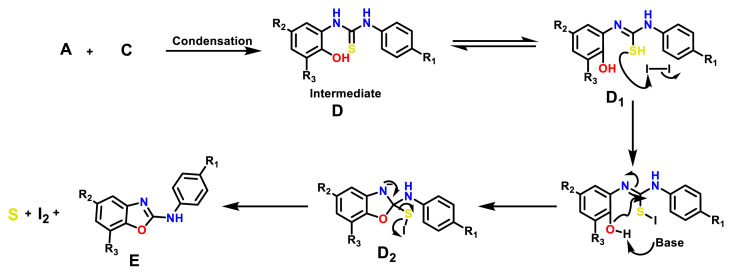
Plausible mechanism for I2-base mediated oxidative C−O benzoxazole formation.

Mechanistic aspects of synthesising 2-Amino-aryl benzoxazole (E) utilising environmentally benign process is explained in Figure 4. Application of molecular iodine in organic synthesis has increased considerably due to its eco-friendly nature and numerous advantages associated with it [44]. Recently, synthesis of 1, 3, 4-oxadiazoles was reported by Pengfei et al*.* via I_2_-mediated oxidative intramolecular C−O bond formation [45, 46].

A plausible reaction mechanism for the formation of N-phenyl-substituted benzoxazol-2-amine (E) is shown below (Figure 4) [44]. The base-promoted oxidative iodination of intermediate D generates an iodide intermediate D_1_. Base promoted cyclization of D_1_, will form a new C−O bond intermediate (D_2_). Finally, the subsequent regeneration of I_2_ and elemental sulphur affords the amino aryl benzoxazole (E) (Scheme 2).

### 3.3. Antimicrobial activity

**Figure 5:**
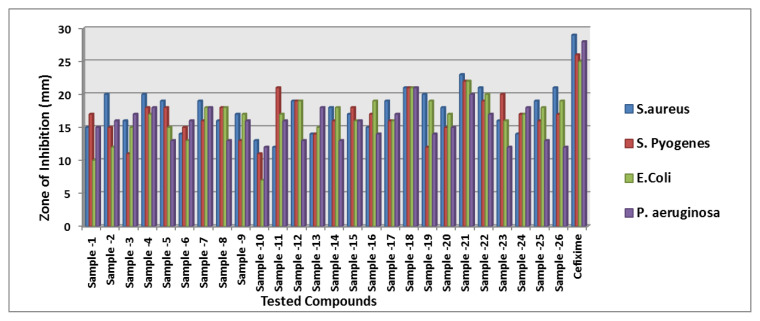
Graphical illustration of in vitro toxicity effect of all compounds against bacteria.

**Figure 6:**
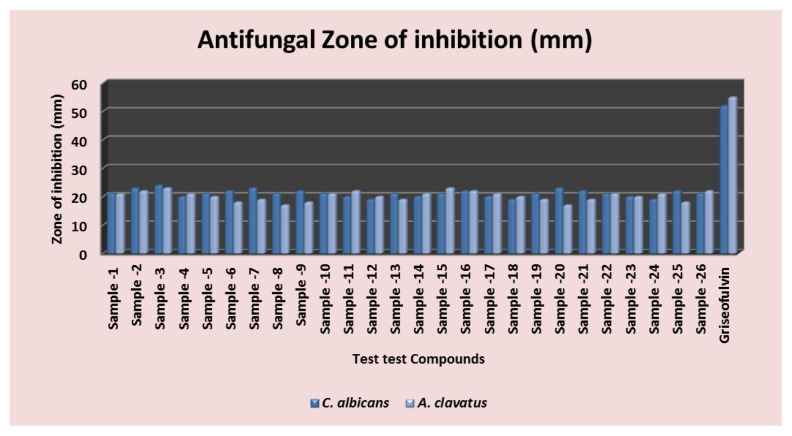
Graphical illustration of in vitro toxicity effect of all compounds against fungi.

In the current research, overall twenty six benzoxazole derivatives from 2-aryl benzoxazoles (14 compounds) and N-phenyl-1,3-benzoxazol-2-amine scaffolds (12 compounds) were screened for their in vitro antifungal and antibacterial potential. Cefixime and griseofulvin were taken as a positive control standard for antibacterial and antifungal studies, respectively. Antimicrobial activity of all the compounds from series 1 and 2 were carried out at the predetermined concentration of 25 μg/mL. Summarised antimicrobial study results were collated in Table 3 and Figures 5 and 6.

#### 3.3.1. Antibacterial activity-Short SAR

All the test compounds from both the series viz. 2-phenyl benzoxazole and 2-Amino phenyl benzoxazoles showed significant antibacterial activity. Among the tested samples, compounds from 2-amino phenyl benzoxazole series showed higher antibacterial potential than 2-phenyl benzoxazole scaffold. Compound-18 showed higher antibacterial activity against *Staphylococcus aureus* (81% inhibition at 25 μg/mL)*.* Both compound-**18** and compound-**21** showed potent activity against *Streptococcus pyogenes* (82%, inhibition) and *staphylococcus aureus (*85% inhibition). Compound-21 showed the highest antibacterial activity against *Escherichia coli* with 90 % of inhibition of growth. Observed inhibition of compound **21** against *pseudomonas aeruginosa* is 72%.

**Figure 7:**
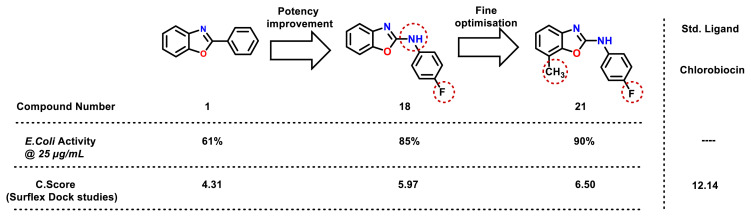
Correlation of *E. coli* in vitro biological activity and surflex docking results for benzoxazole derivatives (Short SAR).

The compound **1** from aryl benzoxazole series showed moderate *E. coli* activity. In order to boost the *E. coli* activity, NH group was inserted between the phenyl and benzoxazole ring. To our surprise, the compound **18** containing an “F” substituent at phenyl ring has shown improved *E. coli* activity. Introduction of a methyl group at 7th position (compound-**21**) further boosted the activity against *E. coli* (Figure 7). Observed biological activities are consistent with the molecular docking studies and consensus score function calculated using surflex docking.

With encouraging results from the disk diffusion studies, two active compounds were evaluated for MIC assay, in order to find the lowest concentration capable of inhibiting the bacterial growth. The MIC values are provided in the Table 4. Both the compounds demonstrated the potential antimicrobial profiles against gram-negative strains (*P. aeruginosa* and *E. coli*) with MICs (approximately 1 μg mL^−^^1^)

#### 3.3.2. Antifungal activity

All the test samples from both the series *viz.* 2-phenyl benzoxazole and 2-amino phenyl benzoxazole series showed significant antifungal activity. Among them, compound-**2**, **3**, and **20** showed good antifungal activity against *Candida albicans* with percentage inhibition of >70%. Furthermore, these compounds showed good antifungal activity against *Aspergillus clavatus* with >70% or higher percentage inhibition.

With encouraging results from the disk diffusion studies, five active compounds were evaluated for MIC assay in order to find the lowest concentration, capable of inhibiting the fungal growth. The MIC values are provided in Table 5. Most of the compounds demonstrated the potential antimicrobial profiles against *Aspergillus clavatus* and *Candida albicans* with MICs (approximately 1 μg mL^−^^1^)

### 3.4. Molecular docking studies

**Figure 8:**
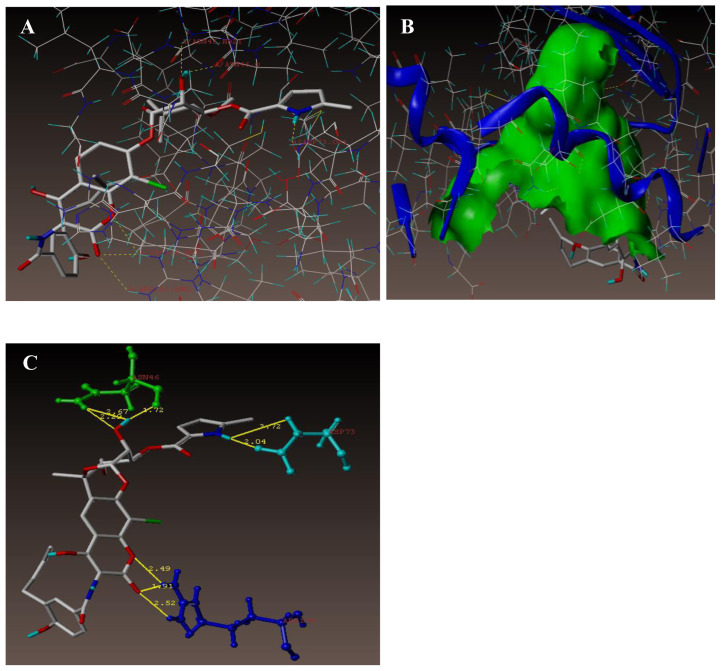
Docked vision of chlorobiocin at the active site of the enzyme PDB: 1KZN.

In order to get the insights on mechanism of antimicrobial activities of the synthesized benzoxazole derivatives, the docking studies and molecular modelling were performed. For the molecular docking studies, X-ray crystal structure of PDB code: 1KZN; resolution 2.30 Å- DNA gyrase *E. coli* 24 kDa domain in complex with clorobiocin was utilised with the help of sybyl-X software’s surflex-dock programme. Clorobiocin is an amino coumarin antibiotic which acts by inhibiting the DNA gyrase enzyme. Binding mode and the “H” bond interactions displayed by clorobiocin is shown in Figures 8A–8C). Chlorobiocin has displayed key hydrogen bonding interactions of ASN46 (1.72 Å; 2.29 Å; 2.67 Å), ASP73 (2.04Å; 3.72 Å), and ARG136 (2.49 Å; 1.91 Å; 2.52 Å).

The binding mode of all the ligands docked in the active site of the enzyme is shown in Figures 8A and 8B. Expected binding energies of the compounds calculated using surflex-dock programme are recorded in Table 6. As per the docking study, all the ligands from both the series have shown very good docking score with respect to *E. coli*.

**Figure 9:**
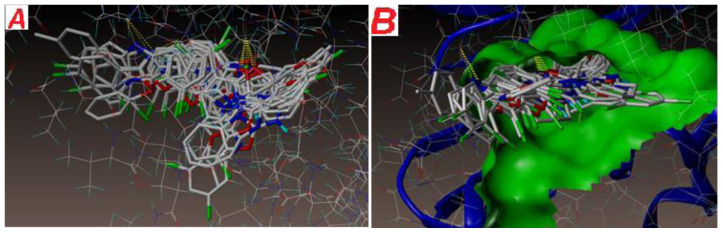
All the compounds at the active site of the enzyme PDB ID: 1KZN- docked view.

Docking poses of each of the ligands were analysed for the key interactions with the protein. For the majority of the compounds, hydrophobic and hydrophilic interactions at active sites of the protein were conserved (ASN46 amino acid residue). Figures 9A and 9B represent docked view of all the compounds at the active site of the DNA gyrase enzyme. Out of all the compounds under investigation, compounds **21** and **18** showed interesting docking outcomes.

**Figure 10:**
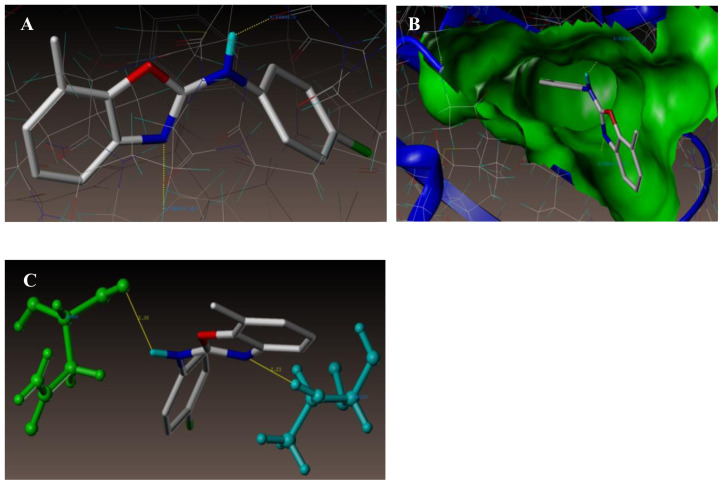
Interface of compound 21 at the binding site of the enzyme (PDB ID: 1KZN).

**Figure 11:**
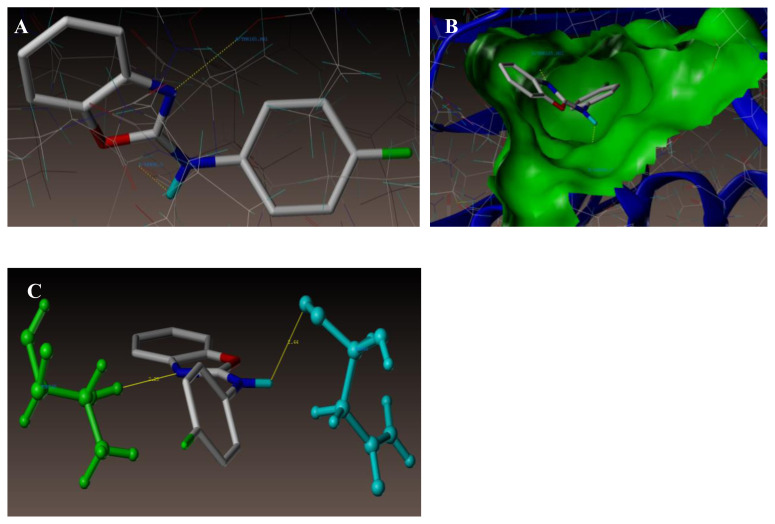
Interface of compound 18 at the binding site of the enzyme (PDB ID: 1KZN).

Figures 10A–10C and Figures 11A–11C represent the 3D views for the compounds 21 and 18, respectively, when docked in the chlorobiocin binding site. The outcome shows that the compounds are well integrated into the binding pocket as in the case of chlorobiocin.

As shown in Figures 10A–10C), at the active site of the enzyme (PDB ID: 1KZN), compound **21** was involved in the two key hydrogen bonding interactions. Hydrogen atom of the amino group in the 2nd position of benzoxazole ring (NH-Ar) and oxygen atom of ASN46 (N-H-----O-ASN46, 2.30 Å) creates a hydrogen bonding interaction. Docked view also showed additional interaction of hydrogen atom of THR165 (N-----H-THR165, 2.23 Å) with nitrogen atom (N) of benzoxazole ring.

As portrayed in Figures 11A–11C, compound **18** at the active site of the enzyme (PDB ID: 1KZN) also exhibited similar pattern of two hydrogen bonding interactions. Hydrogen atom of amino group present on the 2nd position of benzoxazole ring (NH-Ar) and oxygen atom of ASN46 (N-H-----O-ASN46, 2.44 Å) makes a hydrogen bonding interaction. Another interaction came from hydrogen atom of THR165 (N-----H-THR165, 2.25 Å) with nitrogen atom (N) of benzoxazole ring.

**Figure 12:**
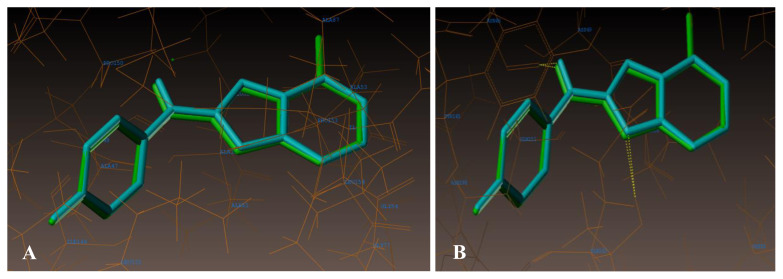
A) Hydrophobic amino acids encircled to compounds 21 (green colour) and 18 (cyan colour). B) Hydrophilic amino acids encircled to compounds 21 and 18.

Figures 12A and 12B signify the additional hydrophilic and hydrophobic amino acids encircled around the studied compounds **21** and **18.**

All the compounds showed a consensus score in the range of 6.50–1.86. Consensus score indicates the summation of all the forces of interface between the protein and ligands calculated via surflex dock program. These scores signify that the molecules are favourably binding to the protein in comparison to the reference chlorobiocin (Table 6). We anticipate the key hydrogen bond interaction of these benzoxazole moieties with ASN46 amino acid residue may be accountable for the consistent antibacterial activity as referred to clorobiocin.

## 4. Conclusion

Synthesis, biological activity and molecular docking studies of two distinct benzoxazole scaffolds are reported in this research work. Eco-friendly synthetic methodologies were utilised for the synthesis of both the scaffolds with quantitative yield. All synthesized benzoxazole derivatives demonstrated substantial antibacterial activity against *Staphylococcus aureus*, *Streptococcus Pyogenes*, *P. Aeruginosa*, and *E. coli* at 25 μg/mL. Furthermore, these compounds showed remarkable antifungal activities against *Aspergillus clavatus* and *Candida albicans* in comparison to the standard griseofulvin. Specifically, the compounds 18 and compound 21 displayed significant antimicrobial potential. Molecular docking studies were executed, to understand the basis of potential antimicrobial activity. Docking experiments revealed that these compounds are interacting in a similar fashion to the known DNA gyrase inhibitor chlorobiocin. Hence, we conclude that benzoxazole derivatives from 2-amino phenyl benzoxazole scaffolds could serve as suitable DNA gyrase inhibitors and may be developed as novel classes of potent antibiotic agents.

## Supporting Information Summary

Following details in support of current research will be found in supporting information.

### Characterization of synthesised compounds

5, 7-dichloro-2-phenylbenzo[d]oxazole (1)

TLC (SiO_2_): Rf = 0.45 (hexane: EtOAc = 10:1). Brown solid, yield=85%, MP: 112–115 °C. FTIR (KBr): 835, 850, 1098, 1242, 1597, 3209 cm^−1^. ^1^H NMR (400 MHz, DMSO-*d*_6_): *δ* 7.66–7.63 (m, 2H), 7.58 (d, 1H), 7.53 (d, 1H), 7.10–7.04 (m, 2H). MS: m/z [M + H] ^+^ calcd for C_13_H_7_Cl_2_NO [M + H] ^+^ 263.9903, found 264.0609.

5, 7-dichloro-2-(p-tolyl) benzo[d]oxazole (2)

TLC (SiO_2_): Rf = 0.46 (hexane: EtOAc = 10:1). Brown solid, yield=87%, MP: 117–120 °C. FTIR (KBr): 838, 860, 1097, 1244, 1598, 3207 cm^−1^. ^1^H NMR (400 MHz, DMSO-*d*_6_): *δ* 8.09–8.06 (m, 2H), 7.95 (d, 1H), 7.46–7.43(m, 2H), 7.38 (d, 1H), 2.29 (s, 3H). MS: m/z [M + H] ^+^ calcd for C_14_H_9_Cl_2_NO [M + H] ^+^ 279.1325, found 280.1506

5, 7-dichloro-2-(2, 6-dichlorophenyl) benzo[d]oxazole (3)

TLC (SiO_2_): Rf = 0.41 (hexane: EtOAc = 10:1). Brown solid, yield=76%, MP: 124–126 °C. FTIR (KBr): 836, 867, 1098, 1245, 1598, 3210 cm^−1^. ^1^H NMR (400 MHz, DMSO-*d*_6_): *δ* 7.91 (d, 1H), 7.65–7.61 (m, 2H), 7.50 (t, 1H), 7.26 (d, 1H). MS: m/z [M + H] ^+^ calcd for C13H5Cl4NO [M + H] ^+^ 332.9954, found 333.9801

7-methyl-2-phenylbenzo[d]oxazole (4) [51]

TLC (SiO_2_): Rf = 0.42 (hexane: EtOAc = 10:1). Light yellow solid, yield=88%, MP: 85–87 °C (lit. 81–83 °C). FTIR (KBr): 835, 1094, 1243, 1597, 3200 cm^−1^. ^1^H NMR (400 MHz, DMSO-*d*_6_): *δ* 8.30 (s, 2H), 7.65 (d, 1H), 7.55–7.52 (m, 3H), 7.28 (d, 1H), 7.14 (d, 1H), 2.64 (s, 3H). MS: m/z [M + H] ^+^ calcd for C_14_H_11_NO [M + H] ^+^ 209.08, found 210.1713.

7-methyl-2-(p-tolyl) benzo[d]oxazole (5) [47]

TLC (SiO_2_): Rf = 0.41 (hexane: EtOAc = 10:1). Yellow solid, yield=82%, MP: 90–95 °C (lit. 88–90 °C). FTIR (KBr): 833, 1097, 1248, 1599, 3210 cm^−1. 1^H-NMR (400 MHz, DMSO-*d*_6_): *δ* = 8.18–8.22 (m, 2 H), 7.59 (d, 1 H), 7.33–7.36 (m, 2 H), 7.24 (dd, 1 H), 7.15 (dd, 1 H), 2.60 (s, 3 H), 2.45 (s, 3 H) ppm. MS: m/z [M + H] ^+^ calcd for C_15_H_13_NO [M + H] ^+^ 223.28, found 224.2920.

2-(2, 6-dichlorophenyl)-7-methylbenzo[d]oxazole (6)

TLC (SiO_2_): Rf = 0.45 (hexane: EtOAc = 10:1). Yellow solid, yield=80%, MP: 114–117 °C. FTIR (KBr): 835, 864, 1093, 1246, 1592, 3201 cm^−1^. ^1^H NMR (400 MHz, DMSO-*d*_6_): *δ* 7.97–7.95 (m, 1H), 7.64–7.60 (m, 2H), 7.49 (t, 1H), 7.27–7.16 (m, 2H), 2.37 (s, 3H). MS: m/z [M + H] ^+^ calcd for C_14_H_9_Cl_2_NO [M + H] ^+^ 279.1332, found 280.0710

7-bromo-2-phenylbenzo[d]oxazole (7) [51]

TLC (SiO_2_): Rf = 0.44 (hexane: EtOAc = 10:1). White solid, yield=86% yield. MP: 117–119 °C (lit. 115–116 °C). FTIR (KBr): 690, 835, 1095, 1245, 1598, 3205 cm^−1^. ^1^H NMR (400 MHz, DMSO-*d*_6_): *δ*= 8.35–8.30 (m, 2H), 7.59–7.53 (m, 5H), 7.28–7.21 (m, 1H); MS: m/z [M + H] ^+^ calcd for C_13_H_8_BrNO [M + H] ^+^ 275.1265, found 278.0558.

7-bromo-2-(p-tolyl) benzo[d]oxazole (8)

TLC (SiO_2_): Rf = 0.43 (hexane: EtOAc = 10:1). Yellow solid, yield=81%, MP: 117–119 °C. FTIR (KBr): 670, 835, 1092, 1243, 1593, 3210 cm^−1^. ^1^H NMR (400 MHz, DMSO-*d*_6_): *δ* 7.96–7.95 (m, 2H), 7.82–7.79 (m, 1H), 7.50–7.48 (m, 2H), 7.39–7.31 (m, 2H), 2.31 (s, 3H). MS: m/z [M + H] ^+^ calcd for C_14_H_10_BrNO [M + H] ^+^ 286.9912, found 286.9865

7-bromo-2-(2, 6-dichlorophenyl) benzo[d]oxazole (9)

TLC (SiO_2_): Rf = 0.47 (hexane: EtOAc = 10:1). Yellow solid, yield=78%, MP: 125–129 °C. FTIR (KBr): 678, 836, 864, 1095, 1246, 1597, 3210 cm^−1^. ^1^H NMR (400 MHz, DMSO-*d*_6_): *δ* 7.96–7.94 (m, 1H), 7.65–7.52 (m, 3H), 7.40–7.30 (m, 2H). MS: m/z [M + H] ^+^ calcd for C_13_H_6_BrCl_2_NO [M + H] ^+^ 343.0014, found 344.0916

2-phenylbenzo[d]oxazole (10) **[**48]

TLC (SiO_2_): Rf = 0.47 (hexane: EtOAc = 10:1). Solid, Yield = 80%, MP = 110–112 °C (lit. 111–113 °C). FTIR (KBr): 836, 1095, 1242, 1598, 3208 cm^−1^. ^1^H NMR (400 MHz, DMSO-*d*_6_) *δ* 8.29–8.25 (m, 2H), 7.83–7.79 (m, 1H), 7.64–7.58 (m, 1H), 7.55–7.53 (m, 3H), 7.38–7.35 (m, 2H). MS: m/z [M + H] ^+^ for C_13_H_9_NO 195.2253 found 196.1821.

2-(p-tolyl) benzo[d]oxazole (11) **[**48]

TLC (SiO_2_): Rf = 0.44 (hexane: EtOAc = 10:1). White solid, Yield=76%. MP: 113–115 °C (lit. 112–114 °C). FTIR (KBr): 837, 1098, 1243, 1599, 3207 cm^−1. 1^H NMR (400 MHz, DMSO-*d*_6_): *δ*= 8.17–8.15 (m, 2H), 7.78–7.76 (m, 1H), 7.59–7.57 (m, 1H), 7.37–7.35 (m, 4H), 2.45 (s, 3H); MS: m/z (ESI) calcd for C_14_H_11_NO [M + H] ^+^ 209.2523, found 211.9318.

2-(2, 6-dichlorophenyl) benzo[d]oxazole (12)

TLC (SiO_2_): Rf = 0.48 (hexane: EtOAc = 10:1). Yellow solid, yield=83%, MP: 115–119 °C. FTIR (KBr): 837, 865, 1093, 1247, 1598, 3205 cm^−1^. ^1^ H NMR (400 MHz, DMSO-*d*_6_): *δ* 8.20–8.17 (m, 2 H), 7.65–7.52 (m, 3H), 7.41–7.35 (m, 2H). MS: m/z [M + H] ^+^ calcd for C_13_H_7_Cl_2_NO [M + H] ^+^ 264.1132, found 266.0421.

5, 7-dichloro-2-(4-chlorophenyl) benzo[d]oxazole (13)

TLC (SiO_2_): Rf = 0.50 (hexane: EtOAc = 10:1). Yellow solid, yield=87%, MP: 118–121 °C. FTIR (KBr): 834, 868, 1095, 1248, 1599, 3210 cm^−1^. ^1^ H NMR (400 MHz, DMSO-*d*_6_): *δ* 8.02 (d, 1H), 7.97–7.76 (m, 2H), 7.83–7.79 (m, 2H), 7.29(d, 1H). MS: m/z [M + H] ^+^ calcd for C_13_H_6_Cl_3_NO [M + H] ^+^ 298.5532, found 298.1216.

2-(4-Chlorophenyl) benzoxazole (14) **[**48]

TLC (SiO_2_): Rf = 0.46 (hexane: EtOAc = 10:1). White solid, yield=85%, mp 145–148 °C (Lit. 144–145 °C); FTIR (KBr): 832, 1093, 1244, 1596, 3110 cm^−1^. ^1^H NMR (400 MHz, DMSO-*d*_6_): *δ* 8.23–8.20 (m, 2H), 7.79–7.75 (m, 1H), 7.61–7.58 (m, 1H), 7.53–7.52 (m, 2H), 7.40–7.37 (m, 2H). MS: m/z [M + H] ^+^ calcd for C_13_H_9_NOCl: 230.0363; found: 230.0370.

5, 7-dichloro-N-(4-fluorophenyl) benzo[d]oxazol-2-amine (15)

TLC (SiO_2_): Rf = 0.48 (Hexane-CH_2_Cl_2_ = 8:2). Brown solid; yield=68%, mp 169–172 °C. FTIR (KBr): 650, 746, 1100, 1271, 1507, 1575, 1600, 1622, 1647, 3049 cm^−1^. ^1^ H NMR (400 MHz, DMSO-*d*_6_): *δ* 8.12(S, 1H), 7.82 (s, 1H), 7.65–7.55 (m, 2H), 7.48 (d, 1H), 7.37 (d, 1H), 7.28–7.22 (m, 2H), 7.19–7.08 (m, 3H). MS: m/z [M + H] ^+^ calcd for C_13_H_7_Cl_2_FN_2_O: 297.1112; found 297.1015.

5, 7-dichloro-N-(4-methoxyphenyl) benzo[d]oxazol-2-amine (16)

TLC (SiO_2_): Rf = 0.38 (Hexane-CH_2_Cl_2_ = 8:2). Light yellow solid; yield=86%, mp 148–150 °C. FTIR (KBr): 650, 746, 1270, 1510, 1573, 1620, 1622, 1650, 2820, 3049 cm^−1^. ^1^ H NMR (400 MHz, DMSO-*d*_6_): *δ* 8.65 (s, 1H), 7.58 (d, 1H), 7.29–7.23 (m, 3H), 6.67–6.63 (m, 2H), 3.74 (s, 3H). MS: m/z [M + H] ^+^ calcd for C_14_H_10_Cl_2_N_2_O_2_: 309.1532; found 309.1021.

5, 7-dichloro-N-phenylbenzo[d]oxazol-2-amine (17)

TLC (SiO_2_): Rf = 0.41 (Hexane-CH_2_Cl_2_ = 8:2). Brown solid, yield=76%, mp 159–160 °C. FTIR (KBr): 650, 746, 1271, 1504, 1573, 1610, 1622, 1647, 3049 cm^−1^. ^1^ H NMR (400 MHz, DMSO-*d*_6_): *δ* 7.95 (s, 1H), 7.58 (d, 1H), 7.53 (d, 1H), 7.49–7.46 (m, 2H), 7.28–7.20(m, 2H), 7.03–6.90 (m, 1H). MS: m/z [M + H] ^+^ calcd for C_13_H_8_Cl_2_N_2_O: 279.1243 Found: 281.0023.

N-(4-fluorophenyl) benzo[d]oxazol-2-amine (18) [49]

TLC (SiO_2_): Rf = 0.47 (Hexane-CH_2_Cl_2_ = 8:2). Yellow solid; yield=88%, mp 168–170 °C (Lit.167–169 °C). FTIR (KBr): 746, 1100, 1271, 1504, 1573, 1600, 1622, 1647, 3049 cm^−1^. ^1^ H NMR (400 MHz, DMSO-*d*_6_): *δ* 7.82 (s, 1H), 7.65–7.55 (m, 2H), 7.48 (d, 1H), 7.37 (d, 1H), 7.28–7.22 (m, 2H), 7.19–7.08 (m, 3H). MS: m/z [M + H] ^+^ calcd for C_13_H_10_FN_2_O: 228.2372; found 229.1662

N-(4-methoxyphenyl) benzo[d]oxazol-2-amine (19) [49]

TLC (SiO_2_): Rf = 0.39 (Hexane-CH_2_Cl_2_ = 8:2). Light yellow solid; yield=80%, mp 138–140 °C (Lit. 137–139 °C). FTIR (KBr): 746, 1270, 1504, 1573, 1620, 1622, 1650, 2820, 3049 cm^−1^. ^1^ H NMR (400 MHz, DMSO-*d*_6_): *δ* 8.79 (s, 1H), 7.55–7.48 (m, 2H), 7.42 (d, 1H), 7.31 (d, 1H), 7.20 (m, 1H), 7.08 (m, 1H), 6.97–6.90 (m, 2H), 3.82 (s, 3H). MS: m/z [M + H] ^+^ calcd for C_14_H_13_N_2_O_2_: 240.2662; found 242.1664.

N-phenylbenzo[d]oxazol-2-amine (20) [33]

TLC (SiO_2_): Rf = 0.42 (Hexane-CH_2_Cl_2_ = 8:2). Yellow solid; yield=83%, mp 125–129 °C. FTIR (KBr): 746, 1270, 1504, 1573, 1625, 1622, 1650, 3049 cm^−1^. ^1^ H NMR (400 MHz, DMSO-*d*_6_): *δ* = 7.39–7.22 (m, 5 H). MS: m/z [M + H] ^+^ calcd for C_13_H_10_N_2_O: 210.2454; found 210.1814.

N-(4-fluorophenyl)-7-methylbenzo[d]oxazol-2-amine (21)

TLC (SiO_2_): Rf = 0.43 (Hexane-CH_2_Cl_2_ = 8:2). Yellow solid; yield=84%, mp 164–167 °C. FTIR (KBr): 746, 1100, 1271, 1504, 1575, 1600, 1630, 1647, 3049 cm^−1^. ^1^ H NMR (400 MHz, DMSO-*d*_6_): *δ* 7.82 (s, 1H), 7.65–7.63 (m, 2H), 7.47–7.44 (m, 1H), 7.38–7.36 (m, 1H), 7.07–6.99 (m, 3H), 2.20 (s, 3H). MS: m/z [M + H] ^+^ calcd for C_14_H_11_FN_2_O: 242.2532; found 243.3721.

N-(4-methoxyphenyl)-7-methylbenzo[d]oxazol-2-amine (22)

TLC (SiO_2_): Rf = 0.38 (Hexane-CH_2_Cl_2_ = 8:2). Light yellow solid; yield=78%, mp 145–147 °C. FTIR (KBr): 746, 1270, 1504, 1573, 1610, 1622, 1650, 2820, 3049 cm^−1^. ^1^ H NMR (400 MHz, DMSO-*d*_6_): *δ* 8.50 (s, 1H), 7.48–7.44 (m, 1H), 7.39–7.36 (m, 1H), 7.26–7.23 (m, 2H), 7.10–7.04 (m, 1H), 6.63–6.61 (m, 2H), 3.74 (s, 3H), 2.20 (s, 3H). MS: m/z [M + H] ^+^ calcd for C_15_H_14_N_2_O_2_: 254.2921; found 254.2720.

7-methyl-N-phenylbenzo[d]oxazol-2-amine (23) [50]

TLC (SiO_2_): Rf = 0.45 (Hexane-CH_2_Cl_2_ = 8:2). Pale brown solid, yield=83%, mp 164–167 °C (Lit. m.p. 163–166 °C). FTIR (KBr): 746, 1271, 1504, 1573, 1600, 1622, 1647, 3049 cm^−1^. ^1^ H NMR (400 MHz, DMSO-*d*_6_): *δ* 7.62 (d, 1H), 7.43–7.32 (m, 3H), 7.18–7.11 (m, 2H), 6.97 (d, 1H), 2.47 (s, 3H). MS: m/z [M + H] ^+^ calcd for C_14_H_12_N_2_O: 224.0951. Found: 224.0854.

7-bromo-N-(4-fluorophenyl) benzo[d]oxazol-2-amine (24)

TLC (SiO_2_): Rf = 0.48 (Hexane-CH_2_Cl_2_ = 8:2). Yellow solid; yield=86%, mp 171–172 °C. FTIR (KBr): 660, 749, 1100, 1271, 1504, 1573, 1600, 1622, 1647, 3049 cm^−1^. ^1^ H NMR (400 MHz, DMSO-*d*_6_): *δ* 7.83 (s, 1H), 7.65–7.63 (m, 2H), 7.57–7.56 (m, 1H), 7.49–7.37 (m, 2H), 7. 05–7.02 (m, 2H). MS: m/z [M + H] ^+^ calcd for C_13_H_8_BrFN_2_O: 305.9823; found 305.9820.

7-bromo-N-(4-methoxyphenyl) benzo[d]oxazol-2-amine (25)

TLC (SiO_2_): Rf = 0.40 (Hexane-CH_2_Cl_2_ = 8:2). Yellow solid; yield=82%, mp 148–150 °C. FTIR (KBr): 655, 746, 1270, 1504, 1573, 1610, 1622, 1650, 2820, 3049 cm^−1^. ^1^ H NMR (400 MHz, DMSO-*d*_6_): *δ* 8.48 (s, 1H), 7.60–7.56 (m, 1H), 7.49–7.36 (m, 2H), 7.26–7.24 (m, 2H), 6.65–6.62 (m, 2H), 3.74 (s, 3H). MS: m/z [M + H] ^+^ calcd for C_14_H_11_BrN_2_O_2_: 319.1642; found 321.0813.

7-bromo-N-phenylbenzo[d]oxazol-2-amine (26)

TLC (SiO_2_): Rf = 0.50 (Hexane-CH_2_Cl_2_ = 8:2). Yellow solid; yield=77%, mp 169–171 °C. FTIR (KBr): 660, 749, 1100, 1271, 1504, 1573, 1600, 1622, 1647, 3049 cm^−1^. ^1^ H NMR (400 MHz, DMSO-*d*_6_): *δ* 7.80 (s, 1H), 7.56–7.53 (m, 1H), 7.50–7.36 (m, 4H), 7.28–7.26 (m, 2H), 7. 05–7.02 (m, 1H). MS: m/z [M + H] ^+^ calcd for C_13_H_9_BrN_2_O: 289.1399; found 289.0932.

## Figures and Tables

**Table 1:** Compounds synthesized under Scheme 1.

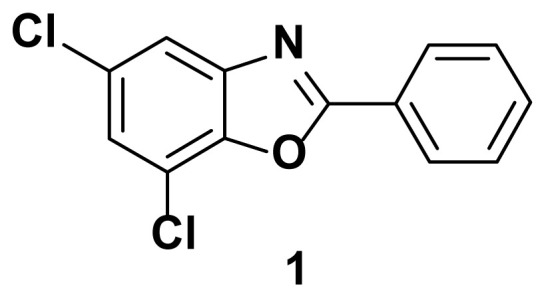	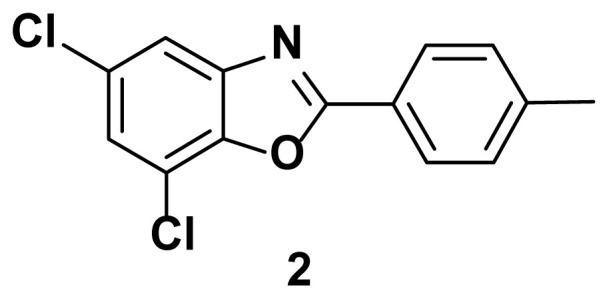	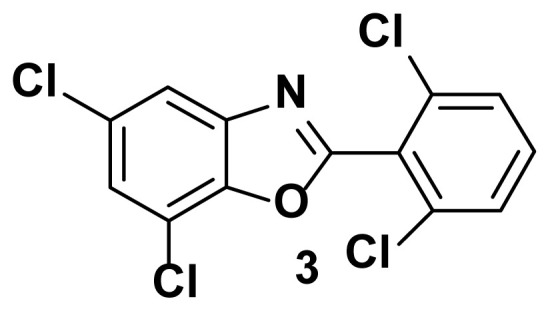
2 h, 82%	3 h, 87%	2.5 h, 76%
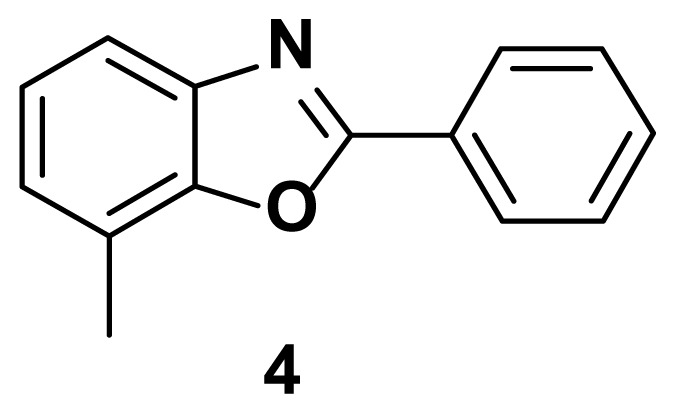	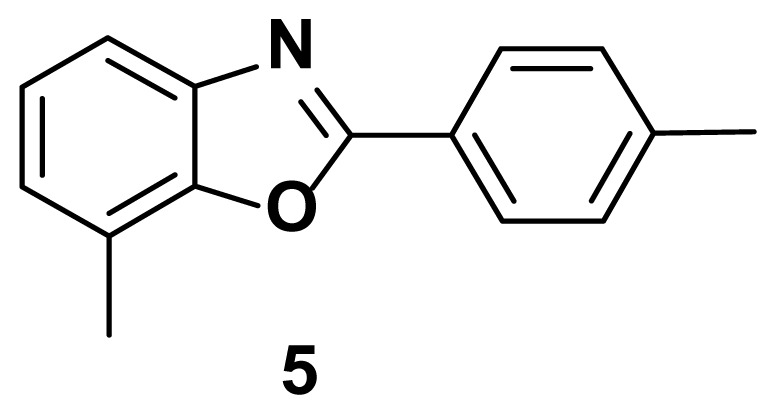	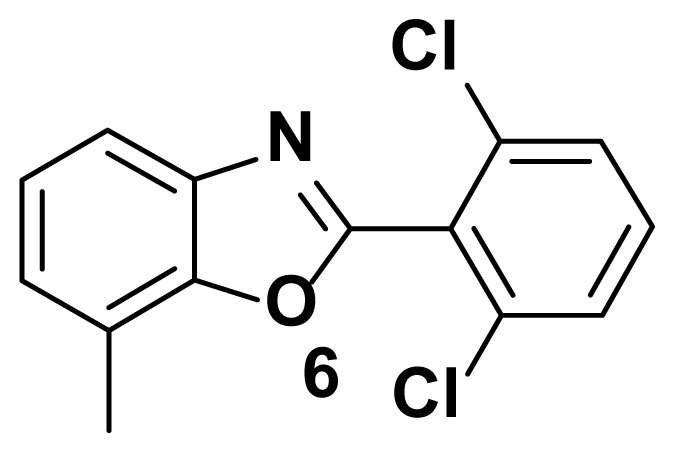
2 h, 88%	1 h, 82%	3 h, 80%
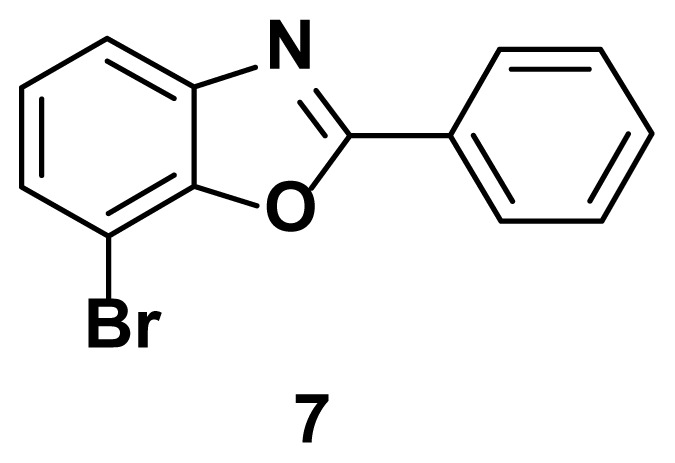	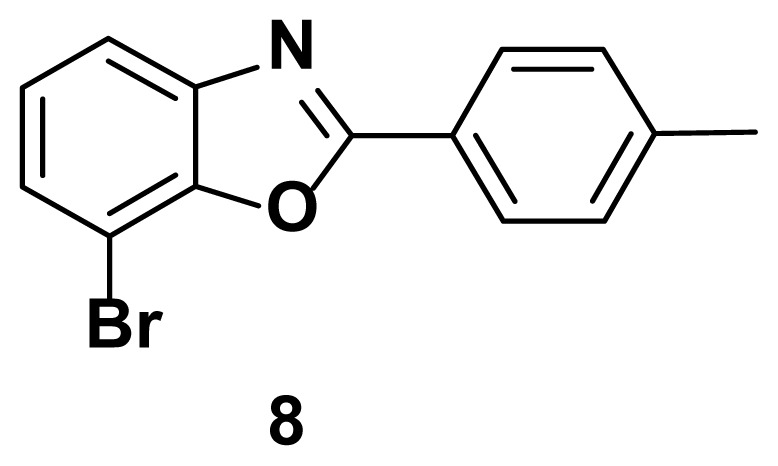	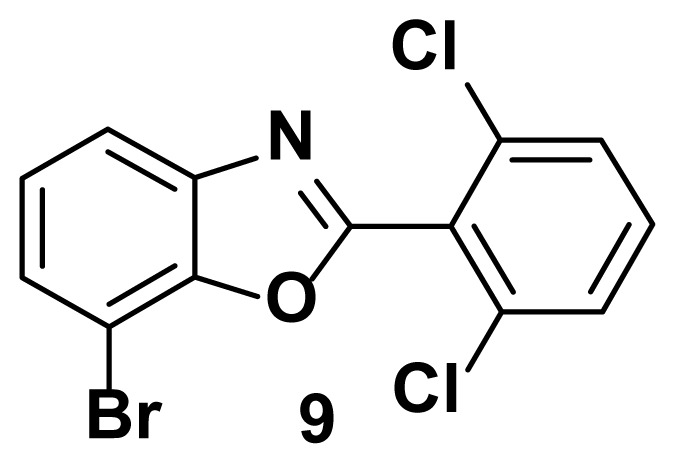
3 h, 86%	2 h, 81%	3 h, 78%
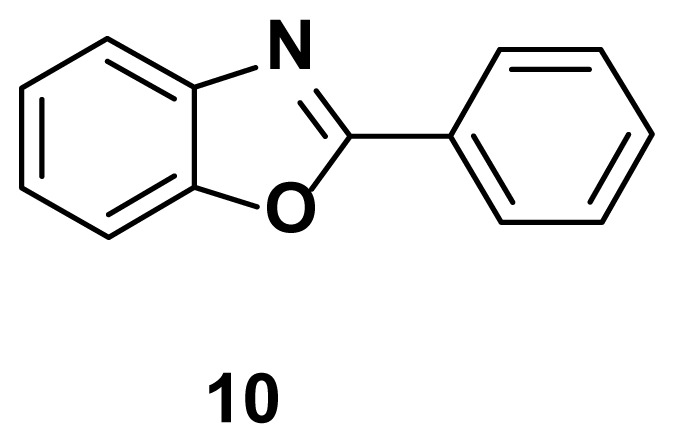	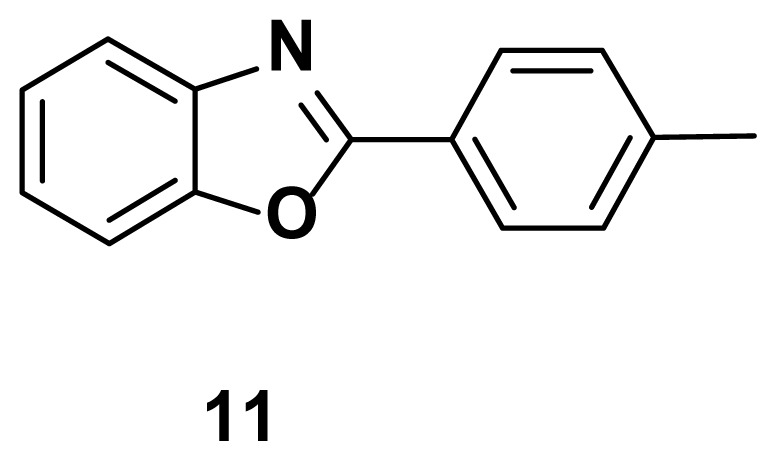	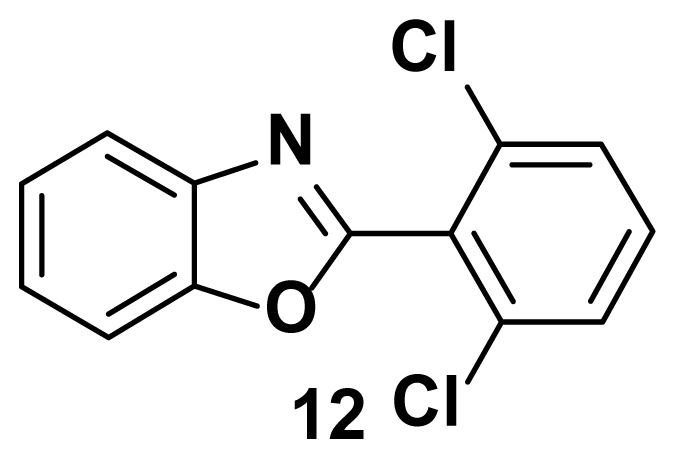
2 h, 80%	3 h, 76%	2.5 h, 83%
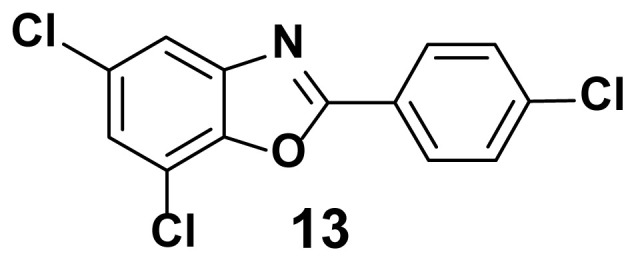	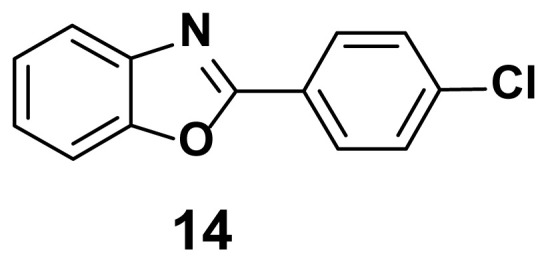	
3 h, 87%	2 h, 85%	

**Table 2:** Compounds synthesized under Scheme 2.

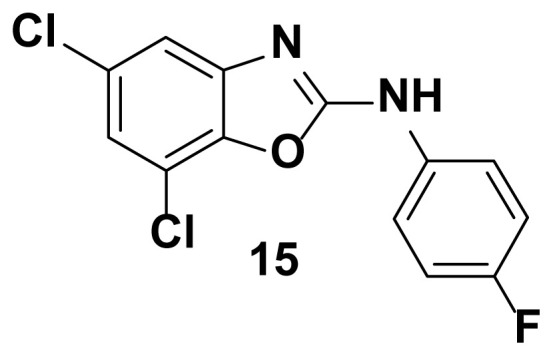	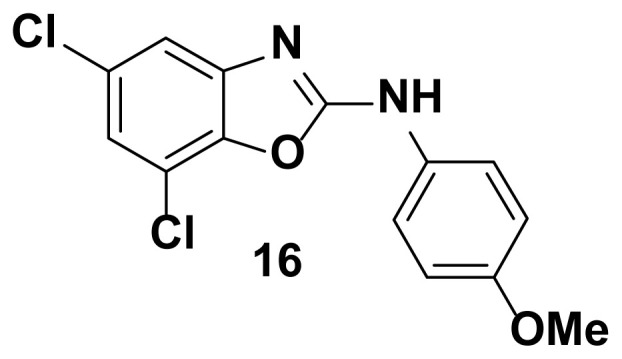	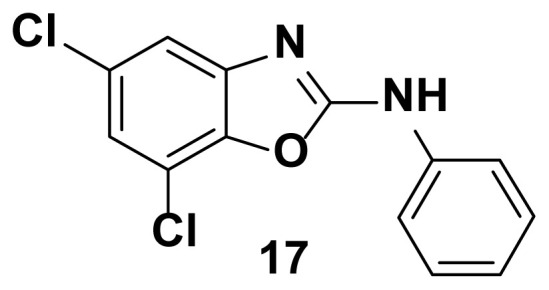
3 h, 68%	2 h, 86%	3 h, 76%
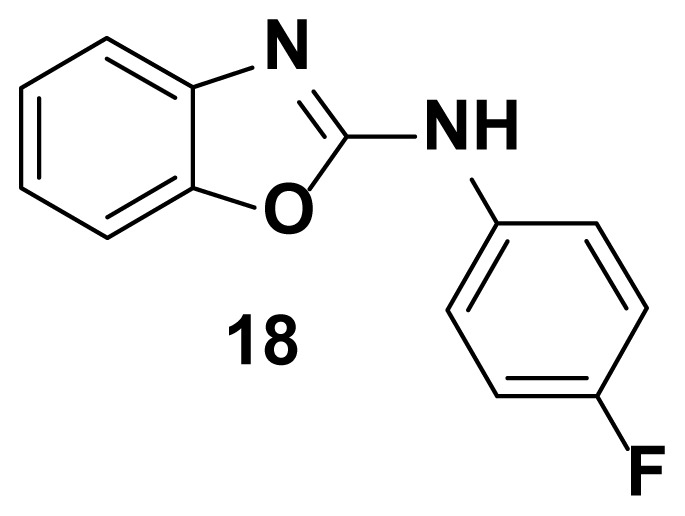	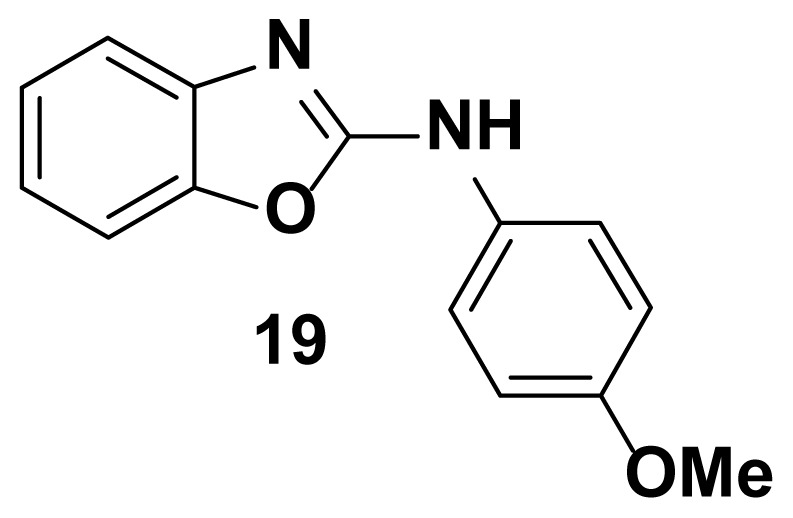	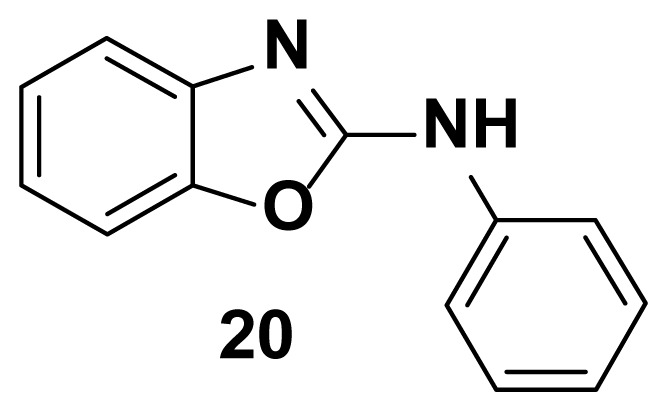
2.5 h, 88%	2 h, 80%	2 h, 83%
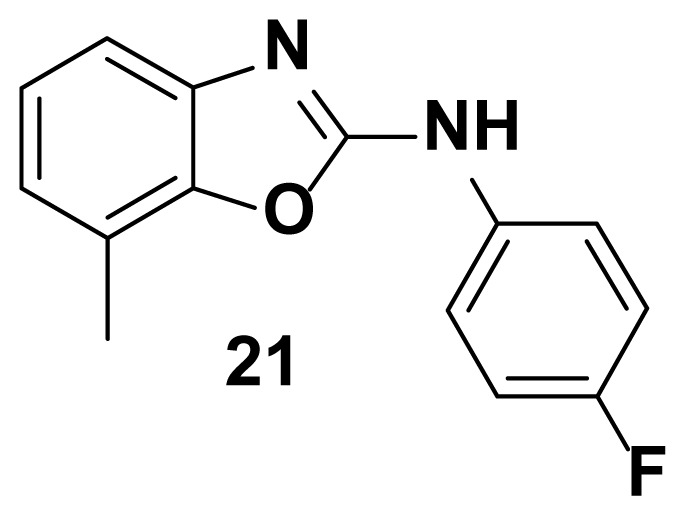	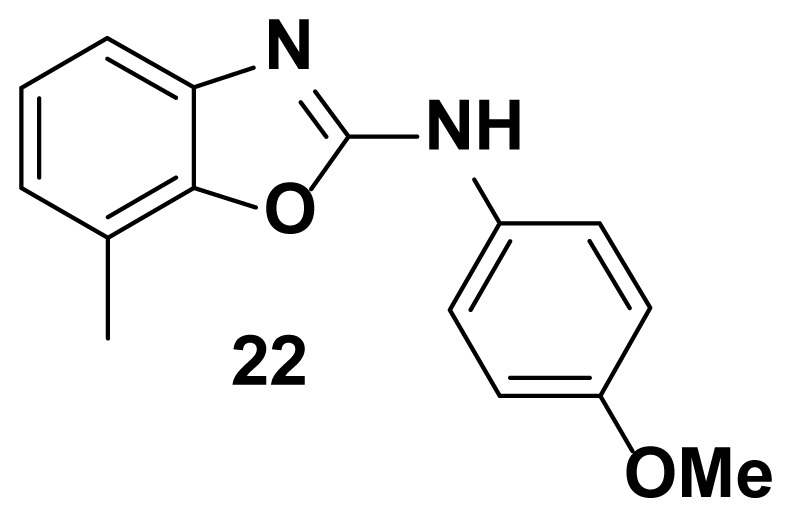	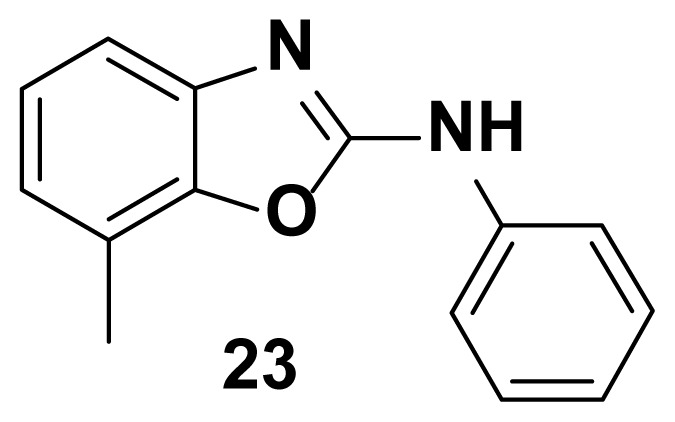
2 h, 84%	3 h, 78%	4 h, 83%
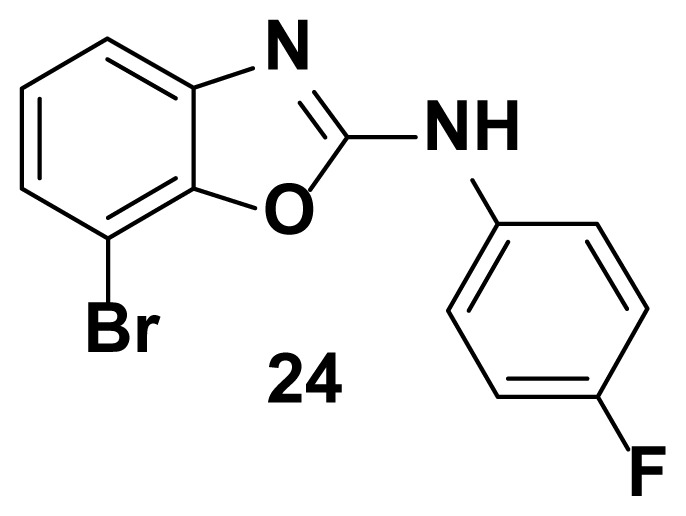	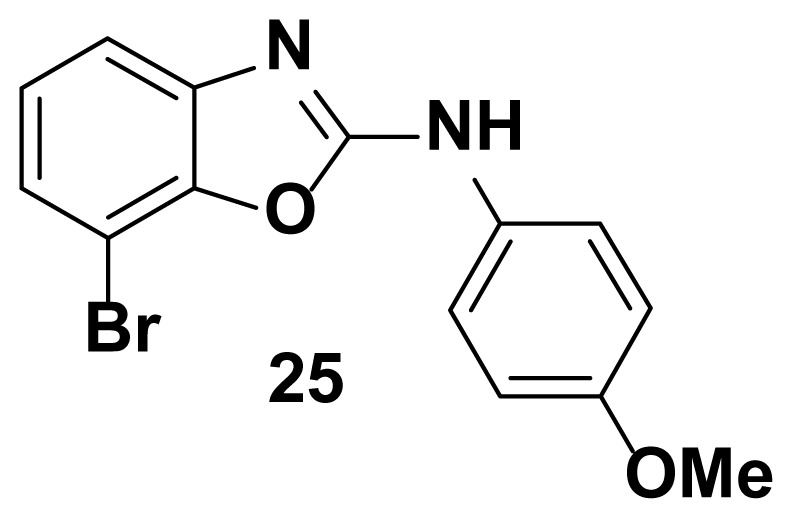	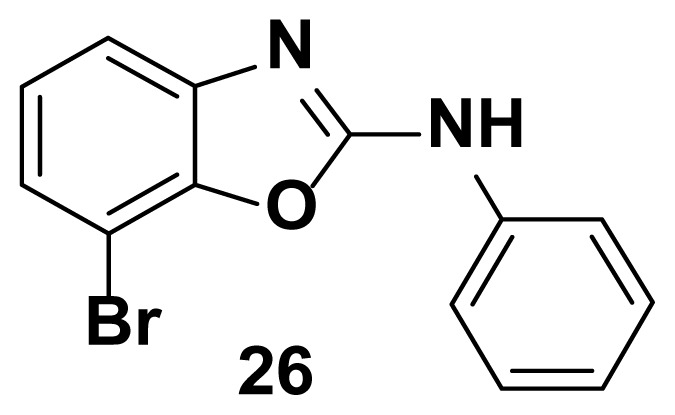
2.5 h, 86%	4 h, 82%	3 h, 77%

**Table 3:** Antibacterial and antifungal activity of samples zone of inhibition in mm) and in percentage.

Compound details	Zone of inhibition in mm and percentage @ 25 μg/mL											
	*S.A mm*	*S.A %*	*S.P mm*	*S.P %*	*E.C mm*	*E.C %*	*P.A mm*	*P. A %*	*C.A mm*	*C. A %*	*A.C mm*	*A.C %*
	Gram-positive bacteria				Gram-negative bacteria				Fungal strains			
	**2-phenyl benzoxazole scaffold**											
Sample -1	15	54%	17	69%	10	40%	15	55%	21	68%	21	68%
Sample -2	20	72%	15	61%	12	50%	16	62%	23	73%	22	70%
Sample -3	16	58%	11	47%	15	63%	17	67%	24	78%	23	73%
Sample -4	20	72%	18	73%	17	61%	18	71%	20	64%	21	68%
Sample -5	19	63%	18	68%	15	61%	13	56%	21	68%	20	64%
Sample -6	14	51%	15	60%	13	55%	16	62%	22	70%	18	55%
Sample -7	19	66%	16	65%	18	63%	18	71%	23	73%	19	59%
Sample -8	16	58%	18	73%	18	63%	13	51%	21	68%	17	52%
Sample -9	17	61%	13	51%	17	61%	16	62%	22	70%	18	55%
Sample -10	13	54%	11	50%	7	35%	12	50%	21	68%	21	68%
Sample -11	12	50%	21	82%	17	61%	16	62%	20	64%	22	70%
Sample -12	19	68%	19	76%	19	66%	13	51%	19	61%	20	64%
Sample -13	14	49%	14	60%	15	70%	18	71%	21	68%	19	59%
Sample -14	18	63%	16	65%	18	72%	13	51%	20	64%	21	68%
	**2-N-phenyl benzoxazole scaffold**											
Sample -15	17	61%	18	73%	16	60%	16	62%	21	68%	23	73%
Sample -16	15	53%	17	67%	19	76%	14	50%	22	70%	22	70%
Sample -17	19	66%	16	65%	16	65%	17	67%	20	64%	21	68%
Sample -18	21	81%	19	82%	20	85%	22	76%	19	61%	20	64%
Sample -19	20	72%	12	47%	19	77%	14	60%	21	68%	19	59%
Sample -20	18	63%	15	61%	17	69%	15	59%	23	73%	17	52%
Sample -21	23	80%	22	85%	22	90%	20	72%	22	70%	19	59%
Sample -22	21	78%	19	76%	20	80%	17	67%	21	68%	21	68%
Sample -23	16	58%	20	79%	16	66%	12	47%	20	64%	20	64%
Sample -24	14	50%	17	69%	17	69%	18	71%	19	61%	21	68%
Sample -25	19	66%	16	65%	18	73%	13	51%	22	70%	18	55%
Sample -26	21	78%	17	69%	19	78%	12	47%	21	68%	22	70%
	**Positive controls**											
Cefixime	22		19		18		20					
	29		26		25		28					
	33		35		36		37					
Griseofulvin									34		35	
									47		43	
									52		55	

CA: *Candida albicans*; AC: *Aspergillus Clavatus.* SA: *Staphylococcus aureus*; SP: *Streptococcus pyrogenes*; EA: *Escherichia coli*; PA: *Pseudomonas aeruinosa*.

**Table 4:** Minimum inhibitory concentrations (MIC50) of two benzoxazole derivatives against four bacteria.

*Compounds*	*MIC_50_ (*μ*g/mL)*			
	*S. aureus*	*S. pyogenes*	*E. coli*	*P. aeruginosa*
***Compound-18***	*15.3 ± 04*	*16.1 ± 02*	*16.5 ± 07*	*16.6 ± 04*
***Compound-21***	*15.2 ± 03*	*14.8 ± 05*	*13.2 ± 01*	*18.7 ± 05*
***Cefixime***	*0.9 ± 0.05*	*0.8 ± 0.06*	*1 ± 0.03*	*0.9 ± 0.04*

**Table 5:** Minimum inhibitory concentrations (MIC50) of two benzoxazole derivatives against two Fungi.

Compounds	MIC_50_ (μg/mL)	
	*C. albicans*	*A. clavatus*
**Compound-2**	17 ± 0.4	NA
**Compound-3**	16 ± 0.3	17 ± 0.3
**Compound-11**	NA	17 ± 0.2
**Compound-20**	15 ± 0.1	NA
**Compound-26**	NA	16 ± 0.3
**Griseofulvin**	1.5 ± 0.2	1.3 ± 0.2

**Table 6:** Surflex docking score (kcal/mol) of the derivatives.

Compounds	C score^a^	Crash score^b^	Polar score^c^	D score^d^	PMF score^e^	G score^f^	Chem score^g^
Chlorobiocin ligand	12.14	−1.21	5.33	−189.513	−76.407	−135.711	−34.514
**21**	6.50	−1.16	1.07	−110.684	−10.157	−179.958	−26.970
**26**	6.37	−1.21	1.04	−109.258	−14.587	−191.905	−26.152
**24**	6.31	−1.21	1.03	−113.284	−13.841	−187.995	−26.039
**20**	6.22	−1.21	1.04	−103.153	−14.226	−182.568	−25.451
**18**	5.97	−1.12	1.06	−107.177	−11.794	−168.904	−25.606
**11**	5.76	−0.47	0.04	−95.768	−16.959	−183.261	−21.980
**10**	5.38	−0.36	0.00	−98.595	−9.572	−169.014	−21.588
**14**	5.33	−0.52	0.68	−94.879	−22.125	−174.047	−21.997
**5**	5.16	−0.54	0.00	−106.419	0.342	−172.461	−24.350
**4**	5.15	−0.81	0.77	−99.238	−14.442	−157.338	−24.249
**22**	5.13	−1.40	0.00	−102.502	−20.543	−168.249	−19.687
**19**	4.94	−0.34	1.13	−84.180	−35.500	−124.692	−18.652
**25**	4.92	−0.48	0.00	−98.863	−30.642	−173.096	−19.423
**17**	4.49	−1.43	1.01	−121.605	−17.512	−190.037	−27.384
**1**	4.31	−1.24	0.97	−110.563	−9.570	−167.539	−27.727
**16**	4.29	−0.55	0.00	−119.135	−12.203	−184.250	−22.019
**8**	4.20	−0.46	0.00	−107.520	−11.136	−167.965	−24.504
**6**	4.11	−1.48	0.10	−121.917	−10.342	−211.169	−25.214
**15**	4.06	−1.51	0.71	−127.579	−14.871	−201.069	−28.226
**7**	3.97	−1.63	0.90	−114.840	−6.916	−171.135	−26.125
**12**	3.91	−0.43	0.04	−76.655	−23.335	−164.867	−17.523
**9**	3.51	−0.60	0.00	−68.726	−18.233	−151.434	−16.096
**23**	3.44	−1.19	0.00	−96.530	5.867	−149.220	−22.389
**2**	2.96	−0.31	0.68	−60.627	−33.274	−132.683	−16.842
**13**	2.15	−1.01	0.01	−76.943	−32.598	−153.307	−17.537
**3**	1.86	−0.97	0.03	−74.997	−31.224	−157.151	−17.272

a Consensus score (CScore) integrates a number of general scoring functions for grading the affinity of ligands bound to the active site of a receptor and reports the output of total score.

b Crash score demonstrates the unfitting penetration into the binding site. Crash scores close to 0 are encouraging. Negative numbers specify penetration.

c Polar score indicates the influence of the polar interactions to the total score. The polar score may be valuable for excluding docking results that make no hydrogen bonds.

d D-score used for van der Waals interactions and charge between the ligand and the protein.

e PMF (potential of mean force) score indicates the Helmholtz free energies of relations for protein-ligand atom pairs.

f G-score shows hydrogen bonding, internal (ligand-ligand), and complex (ligand-protein) energies.

g Chem-score facts for lipophilic contact, H-bonding and rotational entropy, alongside with an intercept term.
